# [Retracted] TIPE2 suppresses atherosclerosis by exerting a protective effect on macrophages via the inhibition of the Akt signaling pathway

**DOI:** 10.3892/etm.2024.12782

**Published:** 2024-12-16

**Authors:** Dan Li, Ying Tan

Exp Ther Med 17:2937–2944, 2019; DOI: 10.3892/etm.2019.7316

Following the publication of this paper, it was drawn to the Editors’ attention by a concerned reader that the majority of the flow cytometric assay data shown in Fig. 1C on p. 2940 were strikingly similar to data that had already appeared in different form in another article written by different authors at different research institutes, which was published in the journal *Cancer Gene Therapy* prior to the submission of this paper to *Experimental and Therapeutic Medicine*.

In view of the fact that the abovementioned data had already apparently been published previously, the Editor of *Experimental and Therapeutic Medicine* has decided that this paper should be retracted from the Journal. The authors were asked for an explanation to account for these concerns, but the Editorial Office did not receive a reply. The Editor apologizes to the readership for any inconvenience caused.

